# Dickkopf Homolog 3 (DKK3) as a Prognostic Marker in Lupus Nephritis: A Prospective Monocentric Experience

**DOI:** 10.3390/jcm11112977

**Published:** 2022-05-25

**Authors:** Savino Sciascia, Alice Barinotti, Massimo Radin, Irene Cecchi, Elisa Menegatti, Edoardo Terzolo, Daniela Rossi, Simone Baldovino, Roberta Fenoglio, Dario Roccatello

**Affiliations:** 1 University Center of Excellence on Nephrologic, Rheumatologic and Rare Diseases with Nephrology and Dialysis Unit and Center of Immuno-Rheumatolgy and Rare Diseases (CMID), Coordinating Center of the Interregional Network for Rare Diseases of Piedmont and Aosta Valley (North-West Italy), San Giovanni Bosco Hub Hospital, 10154 Turin, Italy; alice.barinotti@unito.it (A.B.); massimo.radin@unito.it (M.R.); irene.cecchi@unito.it (I.C.); elisa.menegatti@unito.it (E.M.); edoardo.terzolo@edu.unito.it (E.T.); daniela.rossi@unito.it (D.R.); simone.baldovino@unito.it (S.B.); roberta.fenoglio@unito.it (R.F.); dario.roccatello@unito.it (D.R.); 2 Department of Clinical and Biological Sciences, University of Turin, 10126 Turin, Italy; 3 Department of Clinical and Biological Sciences, School of Specialization of Clinical Pathology, University of Turin, 10126 Turin, Italy

**Keywords:** lupus nephritis, systemic lupus erythematosus, DKK3, kidney

## Abstract

Background: The gold standard for diagnosis of lupus nephritis (LN) is still represented by renal biopsy, and serological prognostic biomarkers are still lacking. Dickkopf homolog-3 (DKK3) has been suggested as a marker of tissue fibrosis in different conditions; however, its role in autoimmune diseases needs to be elucidated. Here, we investigated the prognostic role of DKK3 in systemic lupus erythematosus (SLE) patients with and without LN, assessing its changes in relation to kidney function, flares, and interstitial fibrosis. Methods: Overall, 132 SLE patients (57 with LN) were included and prospectively followed up for at least 36 months. DKK3 was measured in serum at baseline. Biopsies were evaluated for glomerular involvement, interstitial fibrosis, and tubular atrophy. Results: Patients with biopsy-proven LN had significantly higher levels of DKK3 than those without (median [min–max]: 215 ng/mL [81–341] vs. 21.1 ng/mL [1–69], *p* < 0.01). DKK3 levels were associated with prevalent chronic kidney diseases (OR: 4.31 [C.I. 2.01–6.61] per DKK3 doubling, *p* < 0.01), higher chronicity index at biopsy (1.75 [1.51–2.77] per DKK3 doubling, *p* < 0.01), and flares rate (OR: 1.45 [C.I. 1.1–5.71] per DKK3 doubling, *p* < 0.044). Conclusions: While kidney biopsy still represents the gold standard for diagnostic and prognostic assessment in LN, DKK3 could represent an additional prognostic tool to monitor SLE patients and guide therapeutic choices.

## 1. Introduction

Dickkopf-related protein 3 (DKK-3) is a secreted glycoprotein of 38 kDa that belongs to the Dickkopf (DKK) family. These proteins are essential regulators of embryomorphic pathways that are reactivable in response to tissue damage and are involved in epithelial-mesenchymal transition and fibrosis through various molecular mechanisms [1,2,3]. In particular, DKK3 is expressed in different organs, such as the heart, liver, eyes, uterus, embryo, placenta, blood, and pancreatic β cells [4,5,6,7,8,9,10]. DKK3 has been described as a pleiotropic protein implicated in several cellular pathways involved in the cell cycle and differentiation. In detail, DKK3 seems to interfere with the Wnt/β-catenin pathway, which is known to be implicated in the progression of chronic kidney disease (CKD) [11,12,13]. While transient WNT-β-catenin activation has been observed to stimulate tissue regeneration after acute kidney injury (AKI), a sustained WNT-β-catenin signal seems to promote kidney fibrosis in CKD, podocyte injury, and proteinuria [13,14,15,16,17,18].

We need more studies to advance our understanding of the role of DKK3 in autoimmune diseases, including systemic lupus erythematosus (SLE). However, a novel study by Ludwig et al. [4] suggested a further potential role for DKK3 in modulating B lymphocyte survival that can potentially lead to changes in B cell responses. In particular, using a SLE model, they demonstrated that the DKK3 can affect the maturation of B2 cells, B1 cell self-maintenance in the peripheral blood, and the antibody and mediators released by B lymphocytes [4].

SLE pathogenic mechanism is determined by alteration of tolerance towards nuclear autoantigens, which results in a series of interconnected events: lymphoproliferation, polyclonal autoantibody production, immune complex formation, and deposit and systemic inflammation [19,20,21,22,23,24]. Lupus nephritis (LN) is a severe complication of SLE [25,26,27]. Despite great advances in expanding the number of pharmacologic interventions in SLE, LN is still associated with a high rate of morbidity and damage accrual in these patients. Critically, accurate prognostic markers, especially when referring to the amount of interstitial fibrosis, are still lacking in this field of research.

Given this and DKK3’s role in kidney and B cell modulation and tissue regeneration, we aimed to prospectively investigate the value of serum levels of DKK3 in SLE patients with and without LN to assess DKK3 changes in relation to kidney function, rate of flares, and the degree of interstitial fibrosis observed at the kidney biopsy. 

## 2. Materials and Methods

### 2.1. Patients

One-hundred and thirty-two SLE patients, according to the 1997 ACR classification criteria [28], fifty-seven of whom presented a biopsy-proven LN, who attended the University Center of Excellence on Nephrological, Rheumatologic and Rare Diseases (ERK-net, ERN-Reconnect, and RITA-ERN Member) with Nephrology and Dialysis Unit and Center of Immuno-Rheumatology and Rare Diseases (CMID) (San Giovanni Bosco Hospital of Turin, Italy) were included in this study. All consecutive patients with SLE diagnosed between 2015 and 2017 with available specimens in the local biobank were included and prospectively followed. Notably, a kidney biopsy was performed to confirm the diagnosis of LN in all 57 patients with suspected renal involvement. Fifty healthy donors were also enrolled as controls. Blood and urine samples were taken at the time of the kidney biopsy or baseline before any immunosuppressant therapy was started. The patients were followed up for at least 36 months. 

The investigation was performed in agreement with the Declaration of Helsinki principles. The participants signed informed consent. 

Demographic, clinical, laboratory, and therapeutic information were prospectively collected, as shown in Table 1. Collected data included sex, age, serum creatinine (sCr), urinalysis, proteinuria, C-reactive protein (CRP), ESR, hemoglobin, platelets, anti-DNA (ELISA and IF), C3, C4, and ENA; all were routinely measured in patient follow-up. The eGFR was calculated using the CKD-EPI equation [29], and patients were classified based on eGFR into the CKD classification. 

### 2.2. Renal Biopsy

Ultrasound-guided renal biopsies were performed as part of the routine clinical diagnostic practice. The biopsies were screened for glomerular involvement, interstitial fibrosis, and tubular atrophy according to the 2003 International Society of Nephrology/Renal Pathology Society (ISN/RPS) classification [30] and the revised 2018 version [31].

### 2.3. Renal Response and Relapse

The Joint European League Against Rheumatism and European Renal Association–European Dialysis and Transplant Association (EULAR/ERA-EDTA) consensus statement were applied to define the renal response [32]. Complete renal response (CR) has been defined as (a) normal or near-normal (within 10% of normal GFR if previously abnormal) GFR; (b) proteinuria < 0.5 g/24 h; and (c) normal levels of C3/C4 with negative anti-dsDNA antibodies. Partial Response (PR) has been defined as (a) normal or near-normal GFR and (b) a ≥50% decrease in proteinuria to sub-nephrotic levels. Renal flares were defined as a reproducible increase in serum creatinine by ≥30% (or a decrease in GFR by ≥10%) and/or an increase in proteinuria of >0.5 g/24 h when CR was initially achieved, or ≥50% in cases of PR.

### 2.4. Immunological Testing

Serum was prepared within 2 h of collection by double centrifugation at room temperature (2000× *g* for 15 min) and stored in aliquots at −80 °C.

Indirect immunofluorescence on HEp-2 cell substrate (Euroimmun AG, Lübeck, Germany) was performed for antinuclear antibodies (ANA) testing, starting from 1:80 screening dilution of sera up to 1:640, as appropriate. Fluoroscopic patterns were reported according to the International Consensus on Antinuclear Antibody Patterns (ICAP) (www.anapatterns.org, accessed on 1 September 2021).

At first evaluation, anti-dsDNA antibodies were detected using fluorenzymeEliA CTD (connective tissue diseases) screening immunoassay run on Phadia™2500 automated platform (ThermoFisher Scientific, Freiburg, Germany). Anti-dsDNA positive testing was confirmed with Chritidia luciliae immunofluorescence.

Serum for DKK3 testing was collected only at baseline. DKK-3 serum levels were measured using a Human DKK-3 ELISA Kit (Sigma Aldrich, Saint Louis, MI, USA).

### 2.5. Statistical Analyses

Continuous variables are described as mean (S.D.), and categorical variables are presented as numbers (%). Student’s *t*-test and non-parametric Mann–Whitney test were used for normally distributed and non-normally distributed parameters, respectively. Correlations were computed and significance was assessed by Fisher’s test. 

Predictors of flare were investigated by multivariable logistic regression analysis. Kaplan–Meier curves were used to assess the time to renal flare. 

Discriminatory accuracy of biomarkers for LN diagnosis was determined by receiver operating characteristic (ROC) curve analysis and fibrosis scores. The discriminatory power of baseline DKK3 to identify patients at higher risk of progression to CKD after 3-year follow-up was assessed by ROC curve analysis. 

SPSS version 26.0 (IBM, Armonk, NY, USA) and Prism version 8.0 (GraphPad Software, San Diego, CA, USA) software were used. A *p* < 0.05 was considered significant.

## 3. Results

The study enrolled 132 SLE patients with a mean age of 41.7 ± 7.1, 102 out of 132 (77%) were females (Table 1). Focusing on renal involvement, 57 patients out of 132 (43.2%) had biopsy-proven LN. In particular: 46/57 (80.7%) were diagnosed with class IV (+/−V), 6/57 (10.5%) were diagnosed with class III (+/−V), and 5/57 (8.8%) were diagnosed with class V. The other main clinical manifestations of the cohort are described in Table 1. 

**Figure 1:**
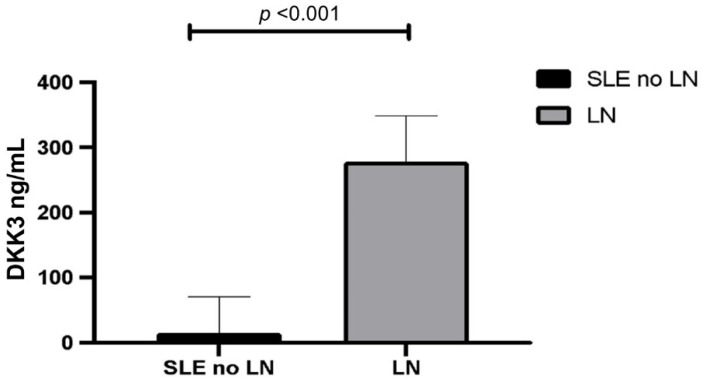
DKK3 levels in SLE with LN vs. SLE with no renal involvement. (SLE = systemic lupus erythematosus; LN = lupus nephritis; DKK3 = Dickkopf-related protein 3).

Patients with LN showed significantly higher serum levels of DKK3 when compared to those without renal involvement (median [min-max] DKK3 levels were 215 ng/mL [81–341] vs. 21.1 ng/mL [1–69] ng/mL, *p* < 0.01, respectively) (Figure 1). 

When evaluated in the 50 healthy controls, the median DKK3 serum values were 10.1 ng/mL [1–13] ng/mL, with detected values ranging around the lower limit of the detectability scale of the immuno-enzymatic test in up to 45% of the cases. 

When focusing on patients with biopsy-proven LN, crude logistic regression analysis showed that DKK3 levels were associated with prevalent chronic kidney diseases (OR: 4.31 [2.01–6.61] per DKK3 doubling, *p* < 0.01), higher chronicity index at the biopsy (OR: 1.75 [1.51–2.77] per DKK3 doubling, *p* < 0.01), and rate of flares (OR: 1.45 [1.1–5.71] per DKK3 doubling, *p* = 0.044). 

**Figure 2:**
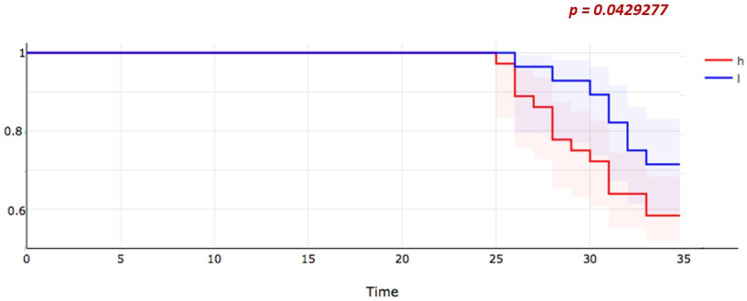
Worsening of CKD stage according to KDIGO classification in patients with LN divided by high or low level at DKK3 at baseline. DKK3 H N = 21 patients; high defined as >75th percentile of the distribution of DKK3 among patients with LN. DKK3 L N = 36 patients, low defined as <75th percentile of the distribution of DKK3 among patients with LN. (CKD = chronic kidney disease; KIDGO = Kidney Disease Improving Global Outcomes; LN = lupus nephritis; DKK3 = Dickkopf-related protein 3).

DKK3 levels were not significantly different when comparing patients according to their renal response after 1 year of observation (*p* = 0.78) (Figure 2).

When evaluating at a 3-year-long clinical follow-up instead, patients who suffered from a worsening in the CKD stage showed significantly more elevated DKK3 levels at baseline than subjects who remained stable or experienced an improvement in the CKD stage. Baseline DKK3 levels seem to be able to identify LN patients more prone to experience a decrease in KDIGO eGFR categories over the 3-year follow-up (Figure 2). 

**Figure 3:**
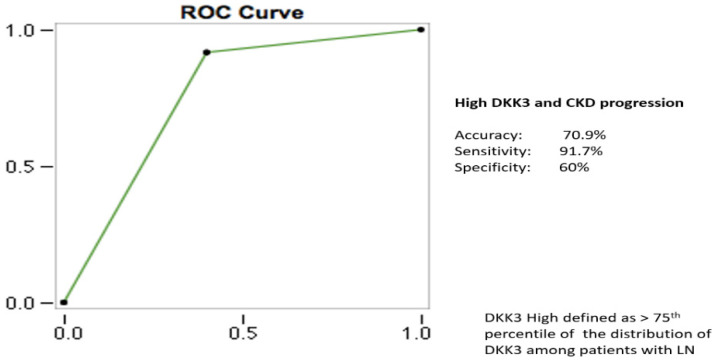
CKD progression as outcome. Characteristics of the ROC curve for the employed DKK3 cut-off. (ROC = receiver operating characteristic; DKK3 = Dickkopf-related protein 3; CKD = chronic kidney disease).

Receiver operating characteristic (ROC) curve analysis was performed for DKK3. Cut-off sensitive analysis was performed using DKK3 levels as a continuous variable using the CKD progression as the outcome (Figure 3). CKD progression was defined as a worsening of the eGFR expressed by the transition from a given KDIGO CKD stage at baseline to a subsequent one during the follow-up (e.g., CKD stage 1 to CKD stage 2). 

The cut-off value > 75th percentile of the distribution amongst LN patients was identified as the one with the best diagnostic accuracy for both CKD progression and moderate/severe renal fibrosis (Table 2). For this cut-off, the calculated performance profile was accuracy as expressed by an AUC of 70.9%, sensitivity of 91.7%, and specificity of 60%. For comparison, the performances of different cut-off values were observed as follows. Using the 65th percentile, we used an AUC of 55.1%, sensitivity of 97.1%, and specificity of 48%. Using the 90th percentile, we used an AUC of 66.1%, sensitivity of 81.1%, and specificity of 66%. 

When comparing baseline values of serum creatinine in SLE patients (dichotomized as abnormal > 1.1 mg/dL, approximation needed considering the small sample size) and nephrotic range proteinuria, DDK3 levels > 75th percentile resulted in having a better diagnostic accuracy for the diagnosis of LN (DKK3: AUC of 94%, sensitivity of 97%, specificity of 88%; sCr: AUC of 55%, sensitivity of 77%, and specificity of 51%; nephrotic range proteinuria: AUC of 68%, sensitivity of 88.3%, and specificity of 57%). Similarly, high levels of DKK3 showed discriminatory power to identify patients with a higher degree of renal fibrosis with an AUC as high as 72% (Table 2). 

## 4. Discussion

The diagnosis of LN is still centered on the renal biopsy, still representing the gold standard in evaluating both the entity and characteristics of glomerular, tubular, vascular, and interstitial involvement [26,33,34,35]. However, regardless of its overall safety, it remains an invasive procedure, and its utility for guiding the treating clinical in support of therapeutic choices is severely limited. One should also consider that patients suffering from SLE have an extremely high degree of heterogeneity, as well as when looking at renal involvement: this is the expression of different immunological abnormalities potentially contributing to the progression towards different clinical and histological phenotypes (e.g., a more fibrotic disease). A growing body of investigations is currently ongoing to assess whether available or upcoming biomarkers could play a role in discriminating across such a clinical heterogeneity and potentially modifying the management of patients with LN. The identification of one or more new biomarkers could theoretically have the potential to guide clinicians in selecting a tailored treatment and approach for SLE patients and improving the rates of morbidity and mortality that still burden this condition [25,33,36,37]. 

In this study, after an initial experience reviewing the current literature on the available biomarkers for characterizing LN [38], we chose to focus our attention on renal fibrosis and test the unknown potential of DKK3, which is emerging as a marker of tissue fibrosis in other diseases and that might represent a further prognostic tool in the management of patients with LN. 

When looking at the results of our study, DKK3 serum levels resulted in being significantly higher in LN patients compared to both SLE patients without renal involvement and to healthy donors, suggesting a high specificity of DKK3 in discriminating SLE renal involvement. Thus, despite how DKK3 can potentially be secreted by a variety of damaged tissues, in this SLE cohort, it appears to be a sensitive finding of LN. 

In addition, our findings are in line with the idea that baseline DKK3 levels (e.g., tested on the same day of renal biopsy) might be an indicator of the burden of fibrosis in LN and represent an additional prognostic tool for the management of these patients.

Interestingly, at least in this cohort of patients with SLE, increased values of DDK3 in the serum at baseline seemed to have better diagnostic accuracy when compared to traditional markers of renal involvement, such as serum creatinine or proteinuria. However, the field of biomarkers in LN is constantly evolving, and the accuracy of DDK3 will require further investigation. More critically, with kidney biopsy still being required for the diagnosis of LN, the added value of testing for DKK3 should be explored as a prognostic tool. In particular, in our cohort, the specificity and sensitivity profile were 60% and 91%, respectively, giving a valuable addition to classical biomarkers, such as creatinine and 24 h proteinuria.

Increased levels of DKK3 detected at baseline were also observed in those patients who experienced a worsening of kidney function over the years, further suggesting a specific role in kidney damage progression. In fact, during the 3-year follow-up, 27% of the LN patients exhibited a decrease in eGFR and an upstaging in the KDIGO classification of CKD, and this LN subpopulation exhibiting disease progression also presented with increased DKK3 serum levels at baseline. 

Some limitations to this study have to be acknowledged. First, as mentioned before, SLE is a highly heterogeneous disease, and this is true also when focusing only on renal involvement that can be different from patient to patient, both when evaluating its severity and kind of manifestation. Second, we measured DKK3 serum levels only at the baseline, and moreover, although its pathogenic involvement in LN seems plausible, additional investigations are needed to clarify its precise role in SLE pathogenesis and, in particular, in LN. Future studies are also needed to investigate if changes in DKK3 levels during follow-up will have any clinical utility. Given this and DKK3 expression in different tissues, we cannot be sure that the increase in DKK3 levels seen in LN patients is strictly kidney-specific, considering the systemic nature of SLE and that this particular population of SLE patients often presents with other comorbidities and a more severe clinical history. Third, the association between DKK3 levels and therapy regimen needs further elucidation in a larger cohort, but it does not appear as a relevant confounding factor in this cohort.

Given all these considerations, while kidney biopsy remains the gold standard for diagnostic and prognostic assessment in LN, if confirmed in a larger cohort, DKK3 could represent an additional useful prognostic tool to monitor patients with SLE and eventually guide therapeutic choices.

## Figures and Tables

**Table 1:** Baseline characteristics of included patients. (ANA = antinuclear antibodies; anti-dsDNA = anti-double stranded DNA antibodies; aPL = antiphospholipid antibodies).

Demographics	
Age, median (25th–75th)	45.3 (19.1–61.3)
Females, *n* (%)	102 (77%)
Disease duration at the time of study inclusion, months, median (25th–75th)	6.3 (1.0–9.0)
**Serology**	
ANA, *n* (%)	132 (100%)
Anti-dsDNA, *n* (%)	107 (81%)
Low complement (C3 and/or C4), *n* (%)	118 (89%)
aPL, *n* (%)	47 (35%)
**Clinical Manifestations**	
Joint involvement, *n* (%)	89 (67%)
Oral aphthosis, *n* (%)	45 (34%)
Photosensitivity, *n* (%)	41 (31%)
Skin involvement, *n* (%)	77 (58%)
Hematological involvement, *n* (%)	57 (43%)
Serositis, *n* (%)	17 (13%)
Renal involvement, *n* (%)	57 (43%)
Nephrotic range proteinuria, *n* (%)	36 (28%)
Class IV (+/−V), *n* (%)	46 (35%)
Class III (+/−V), *n* (%)	6 (5%)
Class V, *n* (%)	5 (4%)

**Table 2:** Diagnostic accuracy of DKK3 defined as dichotomic variable (1 = moderate/severe fibrosis vs. 0 = absent/mild fibrosis). (LN = lupus nephritis; AUC = area under the curve; DKK3 = Dickkopf-related protein 3).

Outcome	AUC	Sensitivity	Specificity
LN diagnosis	94%	97%	88%
Fibrosis scores	72%	90%	63%

## Data Availability

Data can be shared upon request from the corresponding author.
